# SARS-CoV-2 Viral RNA Shedding for More Than 87 Days in an Individual With an Impaired CD8+ T Cell Response

**DOI:** 10.3389/fimmu.2020.618402

**Published:** 2021-01-08

**Authors:** Jackson S. Turner, Aaron Day, Wafaa B. Alsoussi, Zhuoming Liu, Jane A. O’Halloran, Rachel M. Presti, Bruce K. Patterson, Sean P. J. Whelan, Ali H. Ellebedy, Philip A. Mudd

**Affiliations:** ^1^ Department of Pathology and Immunology, Washington University School of Medicine, Saint Louis, MO, United States; ^2^ Department of Emergency Medicine, Washington University School of Medicine, Saint Louis, MO, United States; ^3^ Department of Molecular Microbiology, Washington University School of Medicine, Saint Louis, MO, United States; ^4^ Department of Medicine, Washington University School of Medicine, Saint Louis, MO, United States; ^5^ IncellDX, Menlo Park, CA, United States

**Keywords:** SARS-CoV-2, COVID-19, cellular immunity, CD8+ T cell, CD4+ T cell

## Abstract

Prolonged shedding of viral RNA occurs in some individuals following SARS-CoV-2 infection. We perform comprehensive immunologic evaluation of one individual with prolonged shedding. The case subject recovered from severe COVID-19 and tested positive for SARS-CoV-2 viral RNA repeatedly as many as 87 days after the first positive test, 97 days after symptom onset. The subject did not have any associated rise in anti-Spike protein antibody titers or plasma neutralization activity, arguing against re-infection. This index subject exhibited a profoundly diminished circulating CD8+ T cell population and correspondingly low SARS-CoV-2-specific CD8+ T cell responses when compared with a cohort of other recovering COVID-19 subjects. CD4+ T cell responses and neutralizing antibody responses developed as expected in this individual. Our results demonstrate that detectable viral RNA shedding in the upper airway can occur more than 3 months following infection in some individuals with COVID-19 and suggest that impaired CD8+ T cells may play a role in prolonged viral RNA shedding.

## Introduction

Viral RNA shedding in the upper respiratory tract for more than 20 days following SARS-CoV-2 infection occurs in a small subset of patients. While most scientists and the United States Centers for Disease Control and Prevention do not believe that viral RNA shed in the upper airway more than 20 days after the onset of COVID-19 symptoms remains infectious (1, 2), prolonged viral RNA shedding does correlate with severity of COVID-19 illness and is most frequently observed in elderly individuals and those admitted to the ICU (3, 4).

The presence of detectable viral RNA may suggest ongoing, yet very low levels, of continued viral replication following the resolution of symptoms when patients are no longer infectious. Alternatively, delayed physiologic clearance of non-infectious viral products may be occurring in these individuals who continue to test positive. A recent report demonstrates that at least a small subset of patients with a history of stem-cell transplant, chimeric antigen receptor T cell therapy or lymphoma with persistent viral shedding have virus that can be recovered in cell culture up to 61 days after the onset of symptoms (5). Understanding why some individuals have delayed clearance of infectious virus and others have delayed clearance of viral RNA may be important for determining immunologic correlates of viral clearance that are critical for vaccine development.

## Methods

### Study Design

Subjects were recruited into two prospective observational clinical studies. Recruitment into the studies required testing for COVID-19 and subjects included in this report all tested positive for SARS-CoV-2 viral RNA by an FDA-approved clinical PCR assay. Both studies were approved by the Washington University in Saint Louis Institutional Review Board (Approval #’s 2020 03 085 and 2020 03 186). We collected and documented verbal consent from each enrolled subject in order to conserve personal protective equipment for direct patient care providers and limit exposure risks for research coordinators and study administrative staff related to the collection and maintenance of potentially contaminated written informed consent documents. All subjects enrolled in the study received a written copy of the consent document to keep and subjects enrolled by a legally authorized representative due to respiratory failure necessitating intubation and mechanical ventilation were provided a written copy of the consent document and verbally assented to continued participation in the study following recovery from their illness. The studies comply with the ethical standards of the Helsinki Declaration.

Blood was drawn at the indicated time-points into EDTA-anticoagulated collection tubes and prepared into plasma and peripheral blood mononuclear cells (PBMC) by Ficoll density gradient centrifugation. Plasma was frozen and stored at -80°C. PBMC were washed, counted, resuspended in 90% fetal bovine serum with 10% DMSO and stored in liquid nitrogen.

### Flow Cytometry

Thawed PBMC from subject # WU 350-013 were analyzed by surface flow cytometry as previously described (6). Antibodies in the surface flow-cytometry staining panel included: CD45 Alexa Fluor 532 (clone HI30, Invitrogen), CD3 Alexa Fluor 700 (clone UCHT1, BioLegend), CD4 Spark 685 (clone SK3, BioLegend), CD8 BV421 (clone RPA-T8, BioLegend) and CD19 BV750 (clone HIB19, BioLegend). The panel also included Zombie NIR viability stain (BioLegend). All antibodies were used at pre-titrated optimal staining concentrations.

### Plasma Viral Load Measurement

Plasma viral load measurement was performed as previously described (7). Briefly, viral RNA was extracted from 100 µL or 200 µL of plasma using the QIAamp Viral Mini Kit (Qiagen) and used immediately in the Bio-Rad SARS-CoV-2 ddPCR kit (Bio-Rad). CDC Nucleocapsid gene primers were used. Data were analyzed using QuantaSoft 1.7 and QuantaSoft Analysis Pro 1.0 software. The reproducible limit of detection of the assay is 313 viral copies per mL of plasma (7).

### Plasma Cytokine Quantification

Thirty-five soluble human cytokines, chemokines and growth factors were quantified from frozen plasma using a magnetic cytokine panel (ThermoFisher). The assay was performed according to the manufacturer’s instructions in duplicate and analyzed on a Luminex FLEXMAP 3D instrument.

### Intracellular Cytokine Stain for Antigen-Specific T Cell Responses

Intracellular cytokine staining to detect antigen-specific CD4+ and CD8+ T cells was performed with pools of overlapping 17-mer peptides from the nucleocapsid (N), membrane (M) and Spike (S) proteins of SARS-CoV-2 (USA-WA1/2020 strain) obtained from BEI Resources, NIAID, NIH. Lyophilized peptides were re-suspended in 10% DMSO and water and then pooled. Three pools were evaluated: 1) a combined N and M pool, 90-peptides, 2) S1 [S_1-668_] pool, 94-peptides, 3) S2 [S_659-1273_] pool, 87-peptides. One million PBMC were co-cultured with costimulatory antibodies directed against CD28 and CD49d along with the indicated pooled peptides at a final concentration of 1 µg/mL of each individual peptide, or alternatively with Phorbol 12-myristate 13-acetate (PMA) (InvivoGen) and Ionomycin (InvivoGen) as a positive control or a DMSO/peptide diluent-only negative control. Samples were incubated for 1 hour before the addition of Brefeldin A and monensin (both from BD Biosciences) and then incubated for an additional 5 h. Surface staining was performed followed by fixation in 1% paraformaldehyde, permeablization with a washing buffer supplemented with 0.1% w/v saponin (Sigma) and intracellular staining using fluorescently labeled antibodies directed against cytokine antigens. We used the following antibodies: CD45 Alexa Fluor 532 (clone HI30, Invitrogen), CD3 Alexa Fluor 700 (clone UCHT1, BioLegend), CD4 APC-Cy7 (clone OKT4, BioLegend), CD8 BV421 (clone RPA-T8, BioLegend), IFN-gamma FITC (clone B27, BD Biosciences), TNF-alpha PerCP-Cy5.5 (clone Mab11, BD Biosciences) and IL-2 APC (clone 5344.111, BD Biosciences). The panel also included Zombie NIR viability stain (BioLegend). All antibodies were used at pre-titrated optimal staining concentrations.

Samples were run on a Cytek Aurora spectral flow cytometer using SpectroFlo software (version 2.1.0, Cytek) and unmixed before final analysis using FlowJo software (version 10, BD Biosciences). The mean number of collected live singlet lymphocytes per sample was more than 140,000. Stimulation index was calculated as the frequency of live, singlet CD3+CD4+ (CD4 T cells) positive for any combination of IL-2 and IFN-gamma expression or live, singlet CD3+CD8+ (CD8 T cells) positive for any combination of IFN-gamma, IL-2 or TNF-alpha expression in the stimulated samples divided by the frequency of cytokine positive events in the negative vehicle-only control sample. Figures were prepared in Prism version 8 (GraphPad Software, Inc).

### Spike Receptor Binding Domain ELISA

SARS-CoV-2 S-receptor binding domain (RBD, residues 331−524) was cloned into pFM1.2. The plasmid was transiently transfected into Expi293F cells using ExpiFectamine reagent (Thermo) and cell supernatants were harvested 96h after transfection. SARS-CoV-2 S-RBD was recovered using nickel agarose beads (Goldbio). Ninety-six-well plates (Nunc MaxiSorp; Thermo Fisher Scientific) were coated with 0.1 µg/well recombinant S-RBD in 1X PBS (Gibco) at 4°C overnight. Plates were blocked for 1.5 h at room temperature with blocking solution [1X PBS supplemented with 0.05% Tween-20 (Sigma) and 10% FBS (Corning)]. Plasma samples were diluted 1:30 with blocking solution, serially diluted 3-fold, and incubated for 1 h at room temperature. Plates were washed three times with T-PBS (1X PBS supplemented with 0.05% Tween-20), and 100 µL anti-human IgM-, IgG- or IgA-horseradish peroxidase conjugated antibody (goat polyclonal; Jackson ImmunoResearch) was added to appropriate wells and incubated for 1 h at room temperature. Plates were washed 3 times with T-PBS and 3 times with 1X PBS, and 100 µL peroxidase substrate (SigmaFast o-phenylenediamine dihydrochloride; Sigma) was added to all wells. The reaction was stopped after 5 min using 100 µL 1M hydrochloric acid, and absorbance at 490 nm was read using a microtiter plate reader (BioTek). Areas under the curve were calculated using Prism version 8 (GraphPad Software, Inc).

### Pseudotype VSV Neutralization

Detection of plasma antibodies that can neutralize a pseudotyped VSV virus expressing the SARS-CoV-2 S protein was performed as previously described (8). Briefly, various dilutions of heat-inactivated plasma were incubated with 10^4^ PFU of VSV-SARS-CoV-2-S_△21_ virus for 1 hour at 37°C. Antibody-virus complexes were added to Vero E6 cells and incubated for 7.5 h at 37°C. Cells were then fixed and stained with Hoechst 33342 nuclear stain (Invitrogen). Images were acquired with the InCell 2000 Analyzer (GE Healthcare) automated microscope to visualize nuclei and infected cells (eGFP-positive cells). Images were analyzed in InCell Analyzer 1000 Workstation Software (GE Healthcare) and data were processed using Prism version 8 (GraphPad Software, Inc).

## Results

### Index Clinical Case

**Figure 1:**
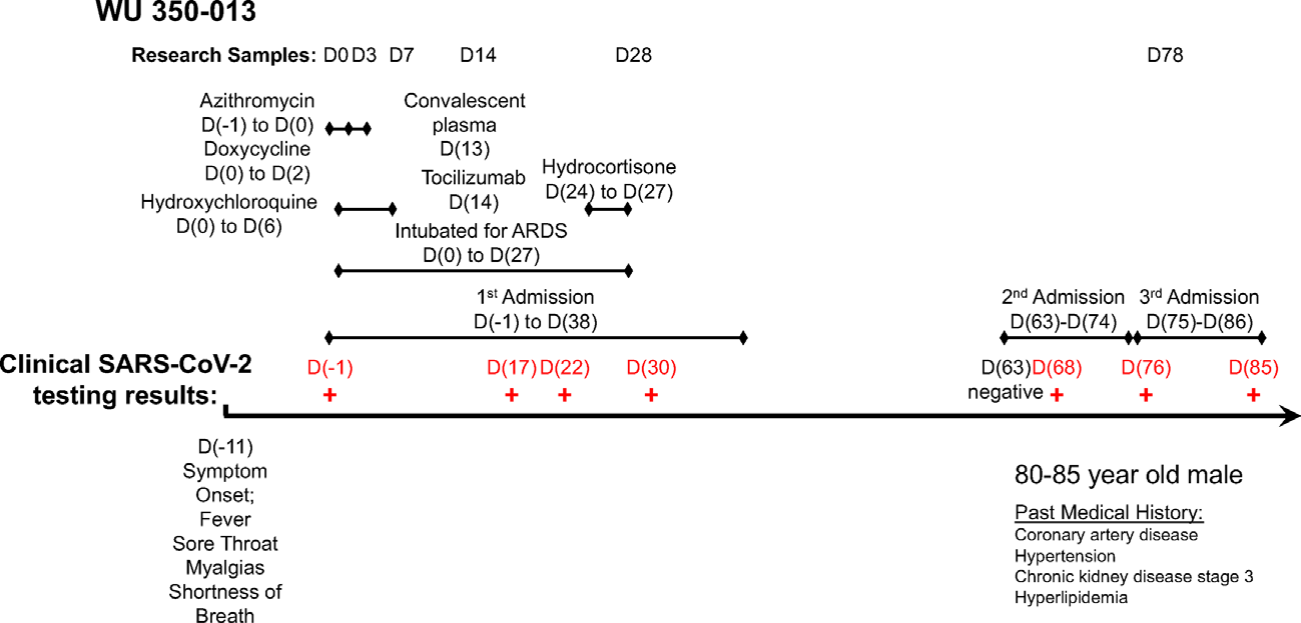
Timeline of study sample collection, administered treatments and clinical SARS-CoV-2 PCR test results for the case subject.

The index subject, WU 350-013, is an 80-85-year-old male with a medical history of coronary artery disease, chronic kidney disease stage 3, hypertension and hyperlipidemia who developed symptoms of a viral respiratory illness 10 days prior to hospital presentation. We enrolled WU 350-013 in the study following return of a positive SARS-CoV-2 clinical PCR test one day after the test was sent to the laboratory for processing. We collected all study samples according to the timeline and protocol established for the study. A timeline of the subject’s course of illness, symptoms of COVID-19, sample collection time-points and administered treatments during hospitalization is presented in **Figure 1**.

WU 350-013 was discharged to a rehabilitation facility following his initial 40-day admission for COVID-19 that included 28 days of mechanical ventilation for acute respiratory distress syndrome. WU 350-013 was then re-admitted to hospital twice for reasons unrelated to COVID-19 25 days and then 37 days after his initial discharge from the hospital (**Figure 1**). The subject was re-tested for COVID-19 during these two admissions on four separate occasions for infection prevention reasons that required COVID-19 testing for all patients admitted to specific areas of the hospital. The last three of these four FDA-approved clinical PCR results returned positive for SARS-CoV-2 viral RNA. The subject was ultimately discharged back to rehabilitation and then to home following the third hospital admission. The patient remained on isolation precautions in his rehabilitation facility, therefore it is felt to be highly unlikely that he was re-exposed to SARS-CoV-2 following his first discharge from the hospital.

### Comprehensive Immunologic Evaluation

**Figure 2:**
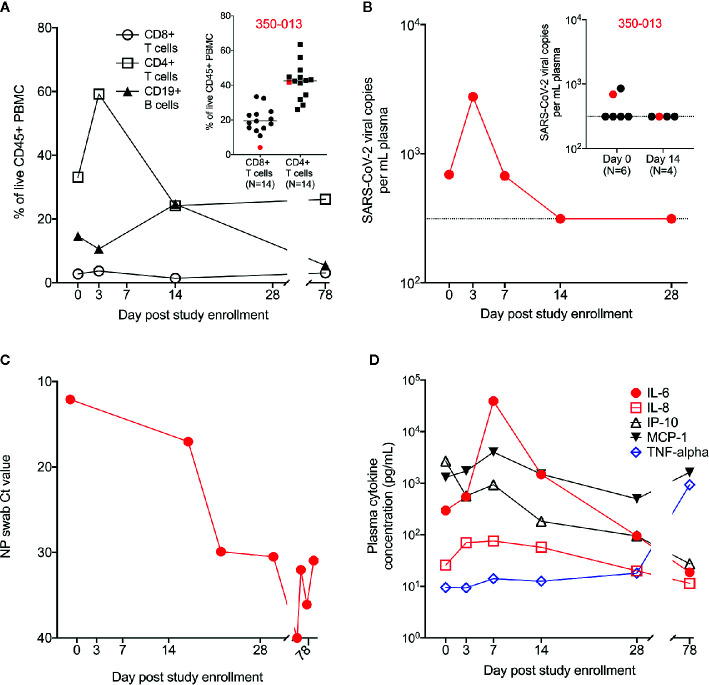
Measurement of cell populations, SARS-CoV-2 viral replication and cytokines over time. **(A)** Frequency of circulating T cell and B cell populations in the PBMC from WU 350-013 at various time-points post-study enrollment. *Inset*: in a separate experiment, the frequency of circulating T cell populations is reported in a cohort of recovering COVID-19 subjects, including a sample from WU 350-013 (denoted in red) at day 78 post-study enrollment. **(B)** WU 350-013 plasma viral load over time. *Inset*: Plasma viral load quantified in WU 350-013 (red) and a cohort of mechanically ventilated ICU subjects at day 0 and day 14 post-study enrollment. Dashed line represents the limit of detection of the assay. **(C)** Cycle threshold or Ct values for clinical nasopharyngeal SARS-CoV-2 testing performed on WU 350-013 throughout the course of the study. Lower Ct values represent a higher burden of viral replication. **(D)** Concentration of select cytokines in plasma from WU 350-013 over time.

We evaluated the subject’s immune response over the course of his acute illness and recovery. We found a profoundly diminished population of CD8+ T cells throughout his illness and recovery as measured by percentage of CD8+ T cells in live CD45+ PBMC, despite normal range values for CD4+ T cells and CD19+ B cells (**Figure 2A**). In a separate experiment, we measured the T cell population size in a cohort of PBMC samples from recovering previously SARS-CoV-2-infected subjects (N=14). Cohort clinical information is reported in **Supplementary Data Table 1**. WU 350-013 had the smallest CD8+ T cell population of the group and his CD4+ T cell population size was at the mean for all recovering patient samples (**Figure 2A** inset).

To better estimate the peak severity of the subject’s illness, we measured SARS-CoV-2 viral replication and circulating plasma cytokines as previously described (7, 9). Plasma viral load peaked on the third day of study enrollment – the subject’s 15^th^ day of symptomatic illness (**Figure 2B**). Plasma viremia was detectable in only a subset of critically ill mechanically ventilated COVID-19 subjects, including WU 350-013 (inset, **Figure 2B**). Viral replication in the airway, as measured by nasopharyngeal viral load, remained high after the resolution of systemic viremia (**Figure 2C**). The peak of systemic viral replication corresponded to the peak of circulating plasma IL-8 and immediately preceded the peak of several inflammatory plasma cytokines, including IL-6 and MCP-1 (**Figure 2D** and **Supplementary Figure 1**). Interestingly, plasma IP-10 concentrations decreased throughout the study interval and plasma TNF-alpha did not substantially increase from relatively low circulating values throughout the course of severe COVID-19. TNF-alpha was elevated at the final day 78 sample collection time-point which appeared to be unrelated to COVID-19 or any respiratory illness and was due to an acute exacerbation of the subject’s heart failure with hypotension that required dobutamine and norepinephrine administration during the third hospitalization.

**Figure 3:**
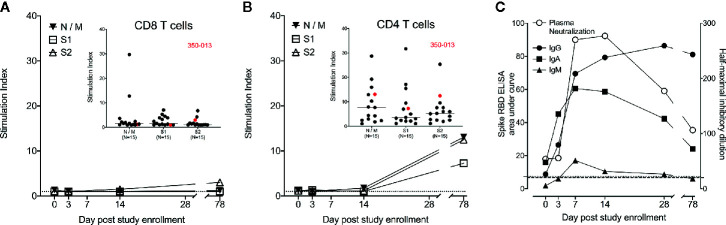
Case subject adaptive immune response over time. **(A)** WU 350-013 SARS-CoV-2-specific CD8+ T cell response to overlapping peptide pools from N, M and S viral proteins as measured by intracellular cytokine staining (ICS) assay at various study time-points. Dotted line is at stimulation index of 1. *Inset*: CD8+ T cell ICS response to peptide pools from the cohort of recovered COVID-19 subjects, including the day 78 post-study enrollment sample from WU 350-013 (red). **(B)** WU 350-013 SARS-CoV-2-specific CD4+ T cell response to overlapping peptide pools from N, M and S viral proteins as measured by ICS at various study time-points. Dotted line is at stimulation index of 1. *Inset*: CD4+ T cell ICS response to peptide pools from recovered COVID-19 subjects, including the day 78 post-study enrollment sample from WU 350-013 (red). **(C)** Development of plasma spike protein receptor binding domain-binding antibodies (left axis, closed symbols) and pseudotyped VSV (expressing SARS-CoV-2 spike protein) neutralizing antibodies in plasma (right axis, open symbols) over time. Light dotted line represents ELISA area under the curve of SARS-CoV-2 negative control plasma; heavy dotted line represents plasma neutralization limit of detection.

Finally, we measured the antigen-specific response in WU 350-013 over time. We assessed SARS-CoV-2 specific CD8+ and CD4+ T cell responses to the Nucleocapsid (N), Membrane (M) and Spike (S) proteins, the three principal targets of CD8+ and CD4+ T cell responses in COVID-19-infected individuals (10). We found no significant SARS-CoV-2 specific CD8+ T cell responses in samples obtained from WU 350-013 prior to the day 78 post-enrollment sample, when WU 350-013 responded just above the median of all other recovering subjects to only the S2 subunit of the spike protein. There were no detectable CD8+ T cell responses to the N, M or S1 antigens (**Figure 3A**). The stimulation index we measured is normalized to the response frequency found within all CD8+ T cells, and WU 350-013 had the lowest number of circulating CD8+ T cells in the cohort, further diluting this single detected S2 response when compared with the other subjects in the cohort. Analysis of background-subtracted frequencies of responding CD8+ T cells was consistent with our analysis of stimulation index (**Supplementary Figure 2**). We observed low antigen-specific CD8+ T cell responses in WU 350-013 despite robust day 78 post-enrollment SARS-CoV-2-specific CD4+ T cell responses in this individual (**Figure 3B**). SARS-CoV-2-specific CD8+ response magnitudes were lower than SARS-CoV-2-specific CD4+ T cell response magnitudes across the cohort of 15 tested subjects (insets **Figure 3**), as others have previously noted (10, 11). WU 350-013’s antigen-specific CD8+ T cell response was below the median value for the N, M and S1 antigens, above the median value for the S2 antigen and the CD4+ T cell response was above the median value for each measured antigen (insets **Figure 3**).

WU 350-013 developed early and robust S-RBD-specific IgG, IgA and IgM antibodies prior to the study sample collected on the 7^th^ day following enrollment, 20 days after the start of symptoms (**Figure 3C**). The appearance of these antibodies corresponded with the appearance of virus neutralizing antibodies in the subject’s plasma (**Figure 3C**). Of note, WU 350-013 developed high-titer neutralizing antibodies by study day 7, six days before the administration of convalescent plasma. Finally, there was no change in the steady rate of decline of antibody responses at the day 78 sample time-point which was taken 10 days after the first of three late-stage positive test results, arguing against a second infection with SARS-CoV-2 prior to the final study sample.

## Discussion

We describe a case of prolonged SARS-CoV-2 viral RNA shedding lasting 87 days after the initial positive clinical PCR test and 97 days after the onset of symptoms. This length of viral RNA shedding is weeks longer than the majority of prolonged viral shedding cases currently reported in the literature (1–4), however, others have recently reported shedding for similar or longer periods of time in a handful of individuals (5, 12). One of these studies has shown that a small number of individuals can shed viable virus that can be cultured *ex vivo* (5). There are no indications in this subject’s immune response that suggest he was re-infected with the virus. Inflammatory cytokines at the latest study time-point did not mirror changes seen during acute infection and antibody titers continued a downward trajectory without suggestion of a secondary antibody response late in the course of the patient’s recovery while clinical SARS-CoV-2 PCR testing remained positive. Furthermore, the clinical nasopharyngeal PCR samples from WU 350-013 maintained high Ct values throughout the later course of the disease correlating with very low viral load in the nasopharynx. Of note, we did not attempt to culture virus from this individual to determine if they harbored replicating virus, but the very high Ct values in the nasopharyngeal samples late in the study suggest that if live virus were present, the risk of transmission would be very low. Therefore, the prolonged shedding interval in this individual does seem most consistent with impaired clearance of viral material rather than ongoing high levels of viral replication. Interestingly, the studied subject exhibited relatively normal appearing anti-SARS-CoV-2 antibody and CD4+ T cell responses but demonstrated severely reduced CD8+ T cell population size throughout his illness and correspondingly low antigen-specific CD8+ T cell responses.

The role of antigen-specific CD8+ T cell responses in COVID-19 has not yet been fully elucidated. We have observed in this study and others have reported that the overall magnitude of the antigen-specific CD8+ T cell response to SARS-CoV-2 is low in individuals recovering from this disease, nearly all of whom successfully clear the virus within 10 to 20 days (10). We and others have also reported upon the depletion of T lymphocytes, specifically CD8+ T lymphocytes, during acute SARS-CoV-2 infection, especially in individuals with critical illness (9, 11, 13). Similar depletions occur in individuals with acute influenza (6, 9) which may affect antigen-specific CD8+ T cell responses in individuals with symptomatic influenza illness (6). Diminished circulating CD8+ T cell population size during acute illness may ultimately impair the host’s ability to generate a large and/or diverse antigen-specific CD8+ T cell response, as suggested by this clinical case. Follow-up study in larger cohorts of patients is needed.

Given the importance of CD8+ T cells in the clearance of viral infections (14, 15), we believe that our observations of such prolonged viral RNA shedding reflect this individual’s uniquely poor CD8+ T cell response during the first three months of his illness. We propose that vaccines which engender SARS-CoV-2-specific CD8+ T cell responses may shorten the interval between viral infection and clearance. Furthermore, the observation that prolonged viral shedding occurs more frequently in individuals with severe illness (4) suggests the possibility that illness severity might also be reduced in individuals with vaccine-enhanced SARS-CoV-2-specific CD8+ T cell responses.

## Data Availability Statement

The original contributions presented in the study are included in the article/**Supplementary Material**. Further inquiries can be directed to the corresponding author.

## Ethics Statement

The studies involving human participants were reviewed and approved by the Washington University in Saint Louis Institutional Review Board. Written informed consent for participation was not required for this study in accordance with national standards and institutional requirements.

## Author Contributions

AD, JO’H, RP, and PM organized the clinical study and actively recruited patients. JT, WA, ZL, BP, and PM performed experiments and analyzed the data. SW, AE, and PM conceived of the study and coordinated the study teams. PM wrote the first draft of the manuscript. All authors contributed to the article and approved the submitted version.

## Funding

This work was supported by a grant from the Barnes Jewish Hospital Foundation and by the Washington University Institute of Clinical and Translational Sciences that is supported by a grant from the ****National Center for Advancing Translational Sciences of the National Institutes of Health**** [****UL1TR002345]. The content is solely the responsibility of the authors and does not necessarily represent the official view of the National Institutes of Health.

## Conflict of Interest

Author BP is employed by the company IncellDX.

The remaining authors declare that the research was conducted in the absence of any commercial or financial relationships that could be construed as a potential conflict of interest.

## Supplementary Material

Supplementary Table 1 Demographic and clinical information from all 15 subjects included in the study.

## References

[1] Wolfel R Corman VM Guggemos W Seilmaier M Zange S Muller MA Virological assessment of hospitalized patients with COVID-2019. Nature (2020) 581(7809):465–9. 10.1038/s41586-020-2196-x 32235945

[2] van Kampen JJ van de Vijver DA Fraaij PL Haagmans BL Lamers MM Okba N Shedding of infectious virus in hospitalized patients with coronavirus disease-2019 (COVID-19): duration and key determinants. Medrxiv [Preprint] (2020). 10.1101/2020.06.08.20125310 PMC7801729 33431879

[3] Wang K Zhang X Sun J Ye J Wang F Hua J Differences of SARS-CoV-2 Shedding Duration in Sputum and Nasopharyngeal Swab Specimens Among Adult Inpatients With COVID-19. Chest (2020) 158(5):1876–84. 10.1016/j.chest.2020.06.015 PMC7305751 32569635

[4] Shi D Wu W Wang Q Xu K Xie J Wu J Clinical characteristics and factors associated with long-term viral excretion in patients with SARS-CoV-2 infection: a single center 28-day study. J Infect Dis (2020) 222(6):910–8. 10.1093/infdis/jiaa388 PMC7337834 32614392

[5] Aydillo T Gonzalez-Reiche AS Aslam S van de Guchte A Khan Z Obla A Shedding of Viable SARS-CoV-2 after Immunosuppressive Therapy for Cancer. N Engl J Med (2020). 10.1056/NEJMc2031670 PMC7722690 33259154

[6] Turner JS Lei T Schmitz AJ Day A Choreño-Parra JA Jiménez-Alvarez L Impaired Cellular Immune Responses During the First Week of Severe Acute Influenza Infection. J Infect Dis (2020) 222(7):1235–44. 10.1093/infdis/jiaa226 PMC7768688 32369589

[7] Patterson BK Seethamraju H Dhody K Corley MJ Kazempour K Lalezari J CCR5 Inhibition in Critical COVID-19 Patients Decreases Inflammatory Cytokines, Increases CD8 T-Cells, and Decreases SARS-CoV2 RNA in Plasma by Day 14. Int J Infect Dis (2020) 103:25–32. 10.1016/j.ijid.2020.10.101 33186704 PMC7654230

[8] Case JB Rothlauf PW Chen RE Liu Z Zhao H Kim AS Neutralizing antibody and soluble ACE2 inhibition of a replication-competent VSV-SARS-CoV-2 and a clinical isolate of SARS-CoV-2. Cell Host Microbe (2020) 28(3):475–85. 10.1016/j.chom.2020.06.021 PMC7332453 32735849

[9] Mudd PA Crawford JC Turner JS Souquette A Reynolds D Bender D Distinct inflammatory profiles distinguish COVID-19 from influenza with limited contributions from cytokine storm. Sci Adv (2020) 6(50):eabe3024. 10.1126/sciadv.abe3024 33187979 PMC7725462

[10] Grifoni A Weiskopf D Ramirez S II Mateus J Dan JM Moderbacher CR Targets of T Cell Responses to SARS-CoV-2 Coronavirus in Humans with COVID-19 Disease and Unexposed Individuals. Cell (2020) 181(7):1489–501. 10.1016/j.cell.2020.05.015 PMC7237901 32473127

[11] Weiskopf D Schmitz KS Raadsen MP Grifoni A Okba NMA Endeman H Phenotype and kinetics of SARS-CoV-2-specific T cells in COVID-19 patients with acute respiratory distress syndrome. Sci Immunol (2020) 5(48):eabd2071. 10.1126/sciimmunol.abd2071 32591408 PMC7319493

[12] Choi B Choudhary MC Regan J Sparks JA Padera RF Qiu X Persistence and Evolution of SARS-CoV-2 in an Immunocompromised Host. N Engl J Med (2020) 383(23):2291–3. 10.1056/NEJMc2031364 PMC7673303 33176080

[13] Schultheiss C Paschold L Simnica D Mohme M Willscher E von Wenserski L Next-Generation Sequencing of T and B Cell Receptor Repertoires from COVID-19 Patients Showed Signatures Associated with Severity of Disease. Immunity (2020) 53(2):442–55. 10.1016/j.immuni.2020.06.024 PMC7324317 32668194

[14] Openshaw PJM Chiu C Culley FJ Johansson C Protective and Harmful Immunity to RSV Infection. Annu Rev Immunol (2017) 35:501–32. 10.1146/annurev-immunol-051116-052206 28226227

[15] Altenburg AF Rimmelzwaan GF de Vries RD Virus-specific T cells as correlate of (cross-)protective immunity against influenza. Vaccine (2015) 33(4):500–6. 10.1016/j.vaccine.2014.11.054 25498210

