# Supplementary material for: SARS-CoV-2 Viral RNA Shedding for More Than 87 Days in an Individual With an Impaired CD8+ T Cell Response
Source: Front Immunol. 2021 Jan 8;11:618402. doi: 10.3389/fimmu.2020.618402 (PMC7820941; doi:10.3389/fimmu.2020.618402)
Supplement: Supplementary file 2 [file DataSheet_1.pdf]

Supplementary Figure 1

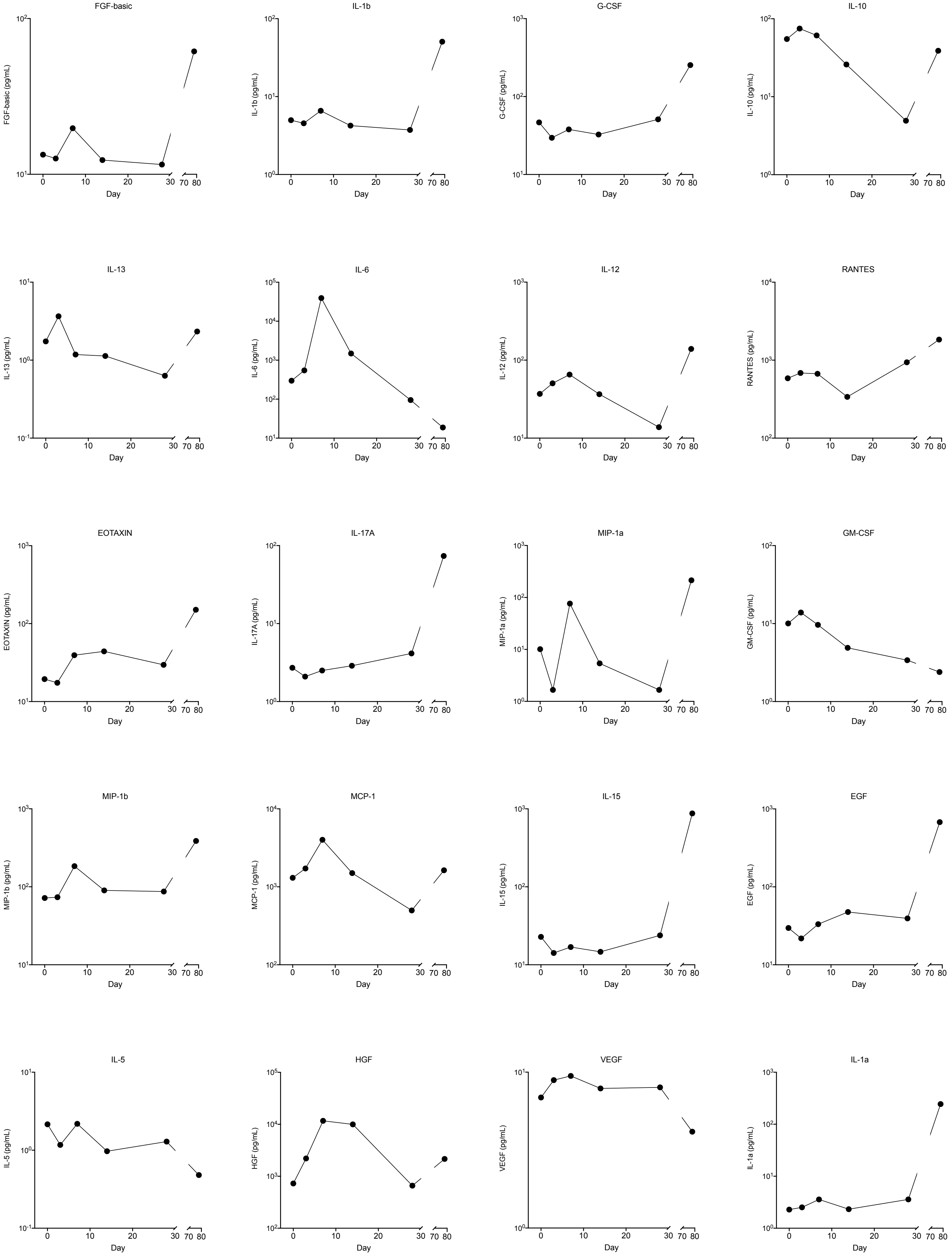

Supplementary Figure 1 - continued

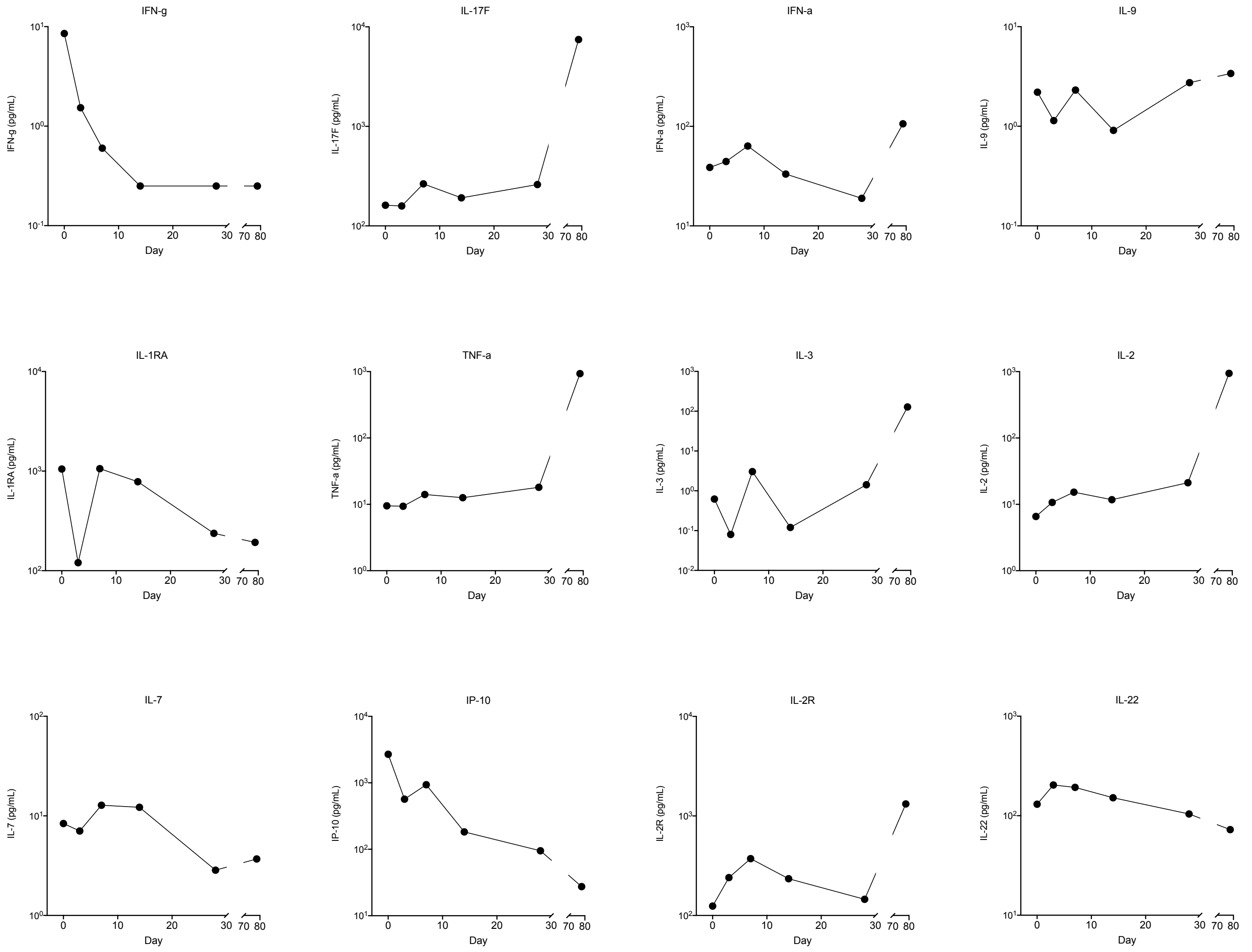

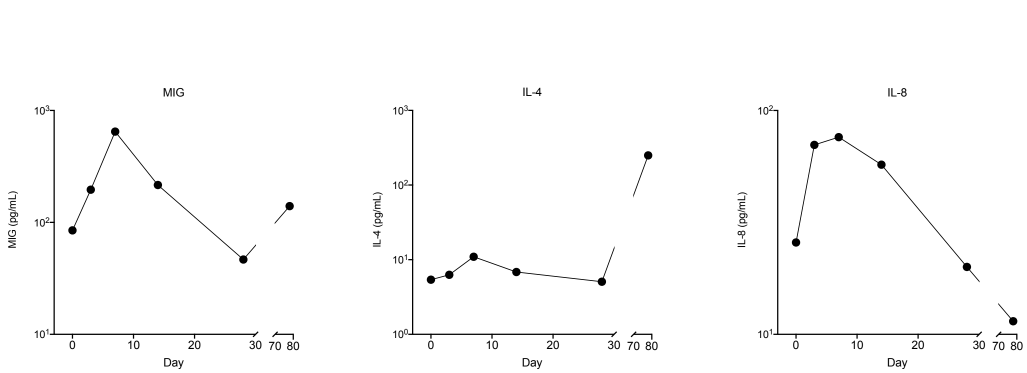

Supplementary Figure 1. Longitudinal values of all 35-individual plasma cytokines measured in WU 350-013 over time. All cytokines are reported in pg/mL.
