# Supplementary material for: SARS-CoV-2 Viral RNA Shedding for More Than 87 Days in an Individual With an Impaired CD8+ T Cell Response
Source: Front Immunol. 2021 Jan 8;11:618402. doi: 10.3389/fimmu.2020.618402 (PMC7820941; doi:10.3389/fimmu.2020.618402)
Supplement: Supplementary file 3 [file DataSheet_2.pdf]

Supplementary Figure 2

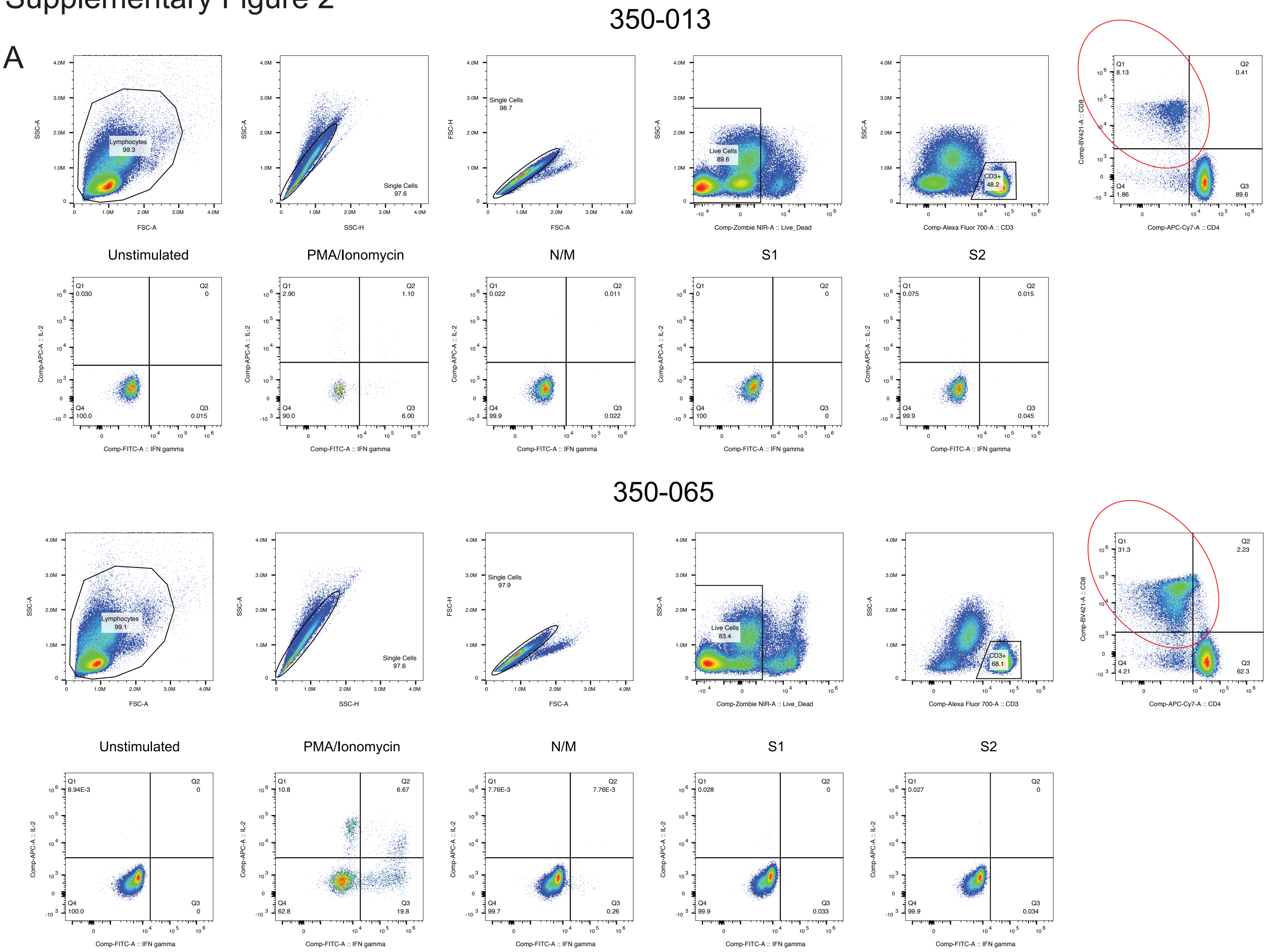

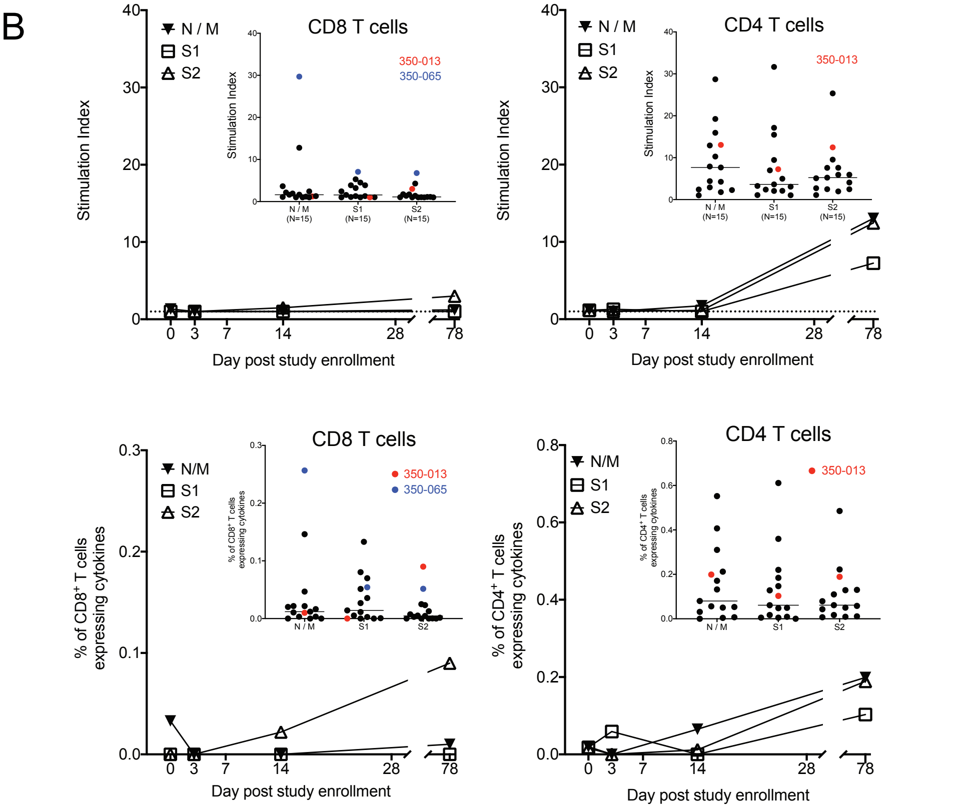

Supplementary Figure 2: Flow cytometry gating strategy and representative plots of intracellular cytokine stain (ICS) data. A) Gating strategies for subjects 350-013 at day 78 and for 350-065. The red circle highlights the significantly diminished CD8+ T cell population in 350-013 when compared to 350-065 and shows the population analyzed in the presented ICS analysis. Representative flow cytometry plots evaluating unstimulated negative control, positive control PMA/ Ionomycin stimulated sample, N/M peptide pool stimulated sample, S1 peptide pool stimulated sample, and S2 peptide pool stimulated samples gated on the CD8+ T cell population. The analysis was performed in FlowJo using a Boolean gating strategy quantifying all combinations of tumor necrosis factor-alpha (TNF-alpha), interferon gamma (IFN gamma) and interleukin-2 (IL-2) positive cells in the CD8+ T cell population, but representative IFN gamma and IL-2 gates are shown. B) Analysis of the data demonstrating similar results between stimulation index and analysis using a strategy that subtracts the total frequency of cytokine-expressing cells from the background unstimulated experimental condition.
